# Contrasting Characteristics and Outcomes of Sports-Related and Non–Sports-Related Traumatic Brain Injury

**DOI:** 10.1001/jamanetworkopen.2023.53318

**Published:** 2024-01-24

**Authors:** Michail Ntikas, William Stewart, Magdalena Ietswaart, Angus M. Hunter, Andrew I. R. Maas, David K. Menon, Lindsay Wilson

**Affiliations:** 1 Division of Psychology, Faculty of Natural Sciences, University of Stirling, Stirling, United Kingdom; 2 Institute of Neuroscience and Psychology, Queen Elizabeth University Hospital, University of Glasgow, Glasgow, United Kingdom; 3 Sport Science, Nottingham Trent University, Nottingham, United Kingdom; 4 Department of Neurosurgery, Antwerp University Hospital and University of Antwerp, Edegem, Belgium; 5 Division of Anaesthesia, University of Cambridge, Cambridge, United Kingdom

## Abstract

**Importance:**

Exposure to traumatic brain injury (TBI) has raised widespread concern over participation in sports, particularly over possible long-term consequences. However, little is known about the outcomes of individuals presenting to hospitals with sports-related TBI.

**Objective:**

To compare the characteristics and outcomes of individuals presenting to hospitals with sports-related and non–sports-related TBI.

**Design, Setting, and Participants:**

The CENTER-TBI (Collaborative European NeuroTrauma Effectiveness Research in TBI) observational cohort study was conducted at hospitals in 18 countries. The study enrolled 4509 patients who had TBI and had an indication for computed tomography (CT), of whom 4360 were 16 years or older. Outcomes were assessed at 3 and 6 months, and groups were compared using regression analyses adjusting for clinical and demographic differences. Data were collected between December 9, 2014, and December 17, 2017, and analyzed from August 2022 to March 2023.

**Exposure:**

Sports-related and non–sports-related TBI with subgroups selected by severity of injury.

**Main Outcomes and Measures:**

The main outcome was the Glasgow Outcome Scale–Extended (GOSE) at 6 months, with secondary outcomes covering postconcussion symptoms, health-related quality of life, and mental health.

**Results:**

A total of 4360 patients were studied, including 256 (6%) with sports-related TBI (mean [SD] age, 38.9 [18.1] years; 161 [63%] male) and 4104 with non–sports-related TBI (mean [SD] age, 51.0 [20.2] years; 2773 [68%] male). Compared with patients with non–sports-related TBI, patients with sports-related TBI were younger, more likely to have tertiary education, more likely to be previously healthy, and less likely to have a major extracranial injury. After adjustment, the groups did not differ in incomplete recovery (GOSE scores <8) at 6 months (odds ratio [OR], 1.27; 95% CI, 0.90-1.78; *P* = .22 for all patients; OR, 1.20; 95% CI, 0.83-1.73; *P* = .34 for those with mild TBI; and OR, 1.19; 95% CI, 0.74-1.92; *P* = .65 for those with mild TBI and negative CT findings). At 6 months, there was incomplete recovery in 103 of 223 patients (46%) with outcomes in the sports-related TBI group, 65 of 168 (39%) in those with mild sports-related TBI, and 30 of 98 (31%) in those with mild sports-related TBI and negative CT findings. In contrast, at 6 months, the sports-related TBI group had lower prevalence of anxiety, depression, posttraumatic stress disorder, and postconcussion symptoms than the non–sports-related group.

**Conclusions and Relevance:**

In this cohort study of 4360 patients with TBI, functional limitations 6 months after injury were common after sports-related TBI, even mild sports-related TBI. Persisting impairment was evident in the sports-related TBI group despite better recovery compared with non–sports-related TBI on measures of mental health and postconcussion symptoms. These findings caution against taking an overoptimistic view of outcomes after sports-related TBI, even if the initial injury appears mild.

## Key Points

**Question:**

Do the outcomes of individuals presenting to hospitals with sports-related traumatic brain injury (TBI) differ from those with non–sports-related TBI?

**Findings:**

This cohort study found that after adjustment for clinical and background differences, individuals with sports-related TBI had better recovery at 6 months of symptoms, mental health, and health-related quality of life than those with non–sports-related TBI. However, there was no significant difference in functional outcomes, and even in individuals with mild sports-related TBI and negative imaging findings, 31% still had some degree of disability.

**Meaning:**

The findings highlight the need for effective clinical follow-up and support for patients who present to hospitals after sports-related TBI irrespective of severity of injury.

## Teaser

This cohort study examines the differences in the characteristics and outcomes of patients with sports-related and non–sports-related traumatic brain injury.

## Introduction

There is increasing attention on the potential brain health consequences of traumatic brain injury (TBI) and repetitive head impacts, in particular their association with increased risk of neurodegenerative disease.^1^ Participation in sports is a common cause of TBI: sports are responsible for approximately 6% of emergency department attendances with TBI^2^ and 20% or more of TBIs recorded in the community as a whole.^3^ Exposure to TBI, both single and repeated, has raised widespread concern over participation in sports, particularly over possible long-term consequences.^4^

Historically, TBI research has often been performed within broad domains, including military or civilian studies, with the latter separated into study of sports-related injuries and hospital-based research that has included mixed etiologies. In this literature, mild sports-related TBI (SR-TBI) has a better prognosis than general mild TBI.^5,6^ However, differences are present between the 2 populations that confound crude comparison: individuals with SR-TBI have lower risk factors for poor outcome than those with general TBI (eg, younger age, fewer preinjury health conditions, and less severe injuries).^6^ Despite the extensive literature on both SR-TBI and general TBI in adults, there are few direct comparisons between sports-related and non–sports-related TBI (NSR-TBI). A recent review^7^ identified only 1 study directly contrasting recovery after SR-TBI and NSR-TBI.^8^ This study used the Rivermead Post-concussion Symptoms Questionnaire (RPQ) at 3 months and found no difference between groups. Therefore, there is a need for work in this area that addresses a range of outcomes and uses data collection over a longer timescale.

Individuals with mild TBI who present to hospitals are a particular concern because of evidence that they frequently experience long-term disability and persisting symptoms.^9,10,11,12^ Approximately half report some limitations in function on the Glasgow Outcome Scale–Extended (GOSE) at 6 months after injury, even those with no abnormality on early computed tomography (CT).^10,13^ A prospective study^14^ found that most individuals with mild TBI or concussion associated with sports and recreation have good recovery from a clinical perspective. However, it has yet to be established whether persisting problems are observed in those who present to hospitals with sports-related injury.

Most hospital-based studies of TBI have not had sufficiently large samples to allow systematic comparisons between patients with different causes of injury. CENTER-TBI (Collaborative European NeuroTrauma Effectiveness Research in TBI) is a large-scale, prospective, longitudinal, observational project that enrolled patients with TBI from 18 countries.^15^ Information is available in the data set concerning the injuries, background factors, clinical course, and outcomes, and this allows SR-TBI to be compared systematically with TBI from other causes. Accordingly, the aim of the study is to identify differences in the demographics and clinical characteristics of SR-TBI and NSR-TBI and compare outcomes up to 6 months after injury. In addition, we examined 2 subgroups created by applying further selection criteria: (1) patients with Glasgow Coma Scale (GCS) scores of 13 to 15 and (2) patients with GCS scores of 13 to 15 and negative CT results. These 2 subgroups represent types of injury of particular interest in the context of SR-TBI. Scores on the GCS of 13 to 15 are often used clinically to define mild TBI, and recent research using this definition had identified patients with persisting problems.^10,13^ Scores on the GCS of 13 to 15 and negative CT results identify an even more mildly injured group, one that fulfills a widely used definition of concussion.^4^

## Methods

The CENTER-TBI core study consists of 4509 patients with data collection between December 9, 2014, and December 17, 2017.^15^ Data were analyzed from August 2022 to March 2023. Ethical approval was obtained for each center in accord with national and local requirements. Details of ethics approvals are on the project website.^16^ Information about race and ethnicity was recorded by investigators at recruitment. We collected this information because it is relevant general background. This cohort study followed the Strengthening the Reporting of Observational Studies in Epidemiology (STROBE) reporting guideline.

### Participants

The criteria for inclusion in the CENTER-TBI project were presentation with TBI within 24 hours of injury, a clinical indication for CT, and availability of informed consent (written consent was obtained at the earliest opportunity, but some patients may have been enrolled initially with oral consent). Patients were excluded if they had a severe preexisting neurologic disorder that would interfere with outcome assessments. Recruitment was to 3 care pathways: emergency department (patients attending the emergency department and discharged), admission (patients admitted to hospital), and intensive care unit (patients admitted to the intensive care unit). For the current analyses, patients younger than 16 years were excluded: this was the age cutoff in the CENTER-TBI project for administering the full set of patient-reported outcomes. Patients were identified as having an SR-TBI if the place of injury was recorded as “sport/recreational” and the cause of injury was not a “road traffic accident” or “violence/attack.” The participant selection process is shown in eFigure 1 in Supplement 1.

### Measures

Demographic and clinical data were collected in the acute stage (Table 1). Preinjury physical health was assessed on the American Society of Anaesthesiologists Physical Status Classification System.^17^ The presence of intracranial abnormalities was recorded from the first CT after injury.^18^ Severity of injury was assessed by the GCS^19^ and the Abbreviated Injury Scale.^20^

**Table 1.** Demographic Characteristics of the Study Participants^a^

Characteristic	SR-TBI (n = 256)	NSR-TBI (n = 4104)
Age, mean (SD), y	38.9 (18.1)	51.0 (20.2)
Sex		
Female	95 (37)	1331 (32)
Male	161 (63)	2773 (68)
Race		
Asian	1 (0.4)	68 (2)
Black	1 (0.4)	61 (2)
White	245 (96)	3777 (92)
Unknown or missing	9 (4)	198 (5)
Highest level of education		
Primary	17 (8)	517 (16)
Secondary	72 (32)	1172 (37)
College or training	134 (60)	1520 (47)
Missing or unknown	33	895
Employment status		
Working (full or part time)	140 (59)	1806 (50)
Not working or homemaker	19 (8)	406 (11)
Retired	26 (10)	1086 (29)
Student	54 (23)	312 (9)
Missing	17	494
Care pathway		
Emergency department	77 (30)	765 (19)
Admission	86 (34)	1376 (34)
Intensive care unit	93 (36)	1963 (48)
Missing	0	0
ASA preinjury physical health		
Healthy	202 (80)	2156 (54)
Not healthy	51 (20)	1815 (46)
Missing	3	133
Preinjury neurologic condition		
Absent	244 (96)	3579 (90)
Present	10 (4)	390 (10)
Missing	2	135
Preinjury psychiatric condition		
Absent	238 (94)	3368 (85)
Present	16 (6)	581 (15)
Missing	2	155
Previous concussion		
Absent	215 (87)	3410 (91)
Present	31 (13)	360 (9)
Missing	10	334
GCS score at baseline		
3-8	34 (14)	928 (24)
9-12	16 (6)	355 (9)
13-14	35 (14)	650 (17)
15	162 (66)	2017 (51)
Missing	9	154
CT abnormality		
Absent	126 (52)	1458 (39)
Present	116 (48)	2248 (61)
Missing or uninterpretable	14	
Major extracranial injury^b^		
Absent	191 (75)	2636 (64)
Present	65 (25)	1468 (36)

Abbreviations: ASA, American Society of Anaesthesiologists; CT, computed tomography; GCS, Glasgow Coma Scale; NSR-TBI, non–sports-related TBI; SR-TBI, sports-related traumatic brain injury.

^a^ Data are presented as number (percentage) of patients unless otherwise indicated.

^b^ Any non–head and neck Abbreviated Injury Scale scores of 3 or higher (serious injury).

### Outcomes

Follow-up for all patients was scheduled for 3 and 6 months either face-to-face or by postal questionnaire or telephone interview. The main outcome was global functional outcome at 6 months, with secondary outcomes covering postconcussion symptoms, health-related quality of life, and mental health.

#### Global Functional Outcome

The GOSE assesses global functional outcome in 8 categories: death, vegetative state, lower severe disability, upper severe disability, lower moderate disability, upper moderate disability, lower good recovery, and upper good recovery.^21^ The GOSE was a composite of interviews and questionnaires that were scored centrally.^22^ Because the vegetative state cannot be identified separately using a questionnaire, this category was combined with lower severe disability. Missing GOSE values were imputed using a multistate model when assessments were available at other time points up to 18 months after injury,^23^ including 14% of 3-month and 16% of 6-month ratings. We used a cutoff value of lower good recovery or less (GOSE score <8) as an indication of incomplete recovery.

The 12-Item Short-Form Health Survey, version 2 (SF-12v2) assesses health-related quality of life.^24^ Two scores were used: the physical component summary (PCS), which provides a measure of functional outcome, and the mental component summary (MCS), which assesses outcome related to aspects of mental health. Outcomes are expressed as T scores (mean [SD], 50 [10]) based on normative data from a 1998 US sample,^24^ with higher scores indicating better quality of life. Scores range from 10 to 65 for the PCS and 8 to 72 for the MCS in the CENTER-TBI sample. T scores less than 40 on the PCS or MCS are considered to indicate significantly impaired health-related quality of life.^24^

#### Mental Health

Symptoms of posttraumatic stress disorder (PTSD) were assessed with the PTSD Checklist for *DSM-5* (PCL-5).^25^ The questionnaire includes 20 symptoms of PTSD based on the *Diagnostic and Statistical Manual of Mental Disorders, Fifth Edition*.^26^ Scores range from 0 to 80, with higher values indicating greater distress. We used a cutoff value of 33 or more as indicating probable PTSD.^27^

The Patient Health Questionnaire (PHQ-9) assesses 9 symptoms of depression.^28^ Total scores range from 0 to 27, with higher values indicating greater emotional distress. A clinical cutoff value of 10 or more was taken to indicate probable depression.^28^

The Generalized Anxiety Disorder–7 (GAD-7) assesses 7 anxiety symptoms.^29^ The total score ranges from 0 to 21, with greater values indicating greater emotional distress. A clinical cutoff value of 8 or more was applied to indicate probable anxiety disorder.^30^

#### Postconcussion Symptoms

The RPQ consists of 16 symptoms commonly reported after mild TBI or concussion.^31^ Total scores range from 0 to 64, with higher scores indicating more severe symptoms. We used a cutoff value of 16 or greater as indicative of clinically significant postconcussion symptoms.^32^

### Statistical Analysis

To ascertain whether background and clinical variables were independently associated with SR-TBI, we performed binary logistic regression with SR-TBI group membership as the dependent variable and demographic, socioeconomic, and clinical factors as independent variables. When comparing outcomes between patients with SR-TBI and NSR-TBI, we considered first the whole sample and then each of the 2 progressively less injured subsamples. All available data at each time point were used for the primary analyses. The denominator used for calculating percentages of impairment was the number of available outcomes for the assessment. In a supplementary sensitivity analysis, we included only patients with outcomes at both 3 and 6 months. Binary logistic regressions were performed with each of the dichotomized outcome measures. Covariates included were age, sex, highest level of education, preinjury employment status, TBI severity, CT abnormality, major extracranial injury, American Society of Anaesthesiologists Physical Status class, neurologic medical history, psychiatric medical history, and history of concussion (details on covariate levels can be found in Table 1). For missing data on covariates, we used multiple imputation with multivariate imputation by chained equations^33^ in R statistical software.^34^ The number of imputations was 10, and the maximum number of iterations was 5. We controlled for multiple comparisons among the 14 outcome measures by adjusting for the false discovery rate (FDR) using the sequential Bonferroni type procedure as described by Benjamini and Hochberg.^35^ Statistical significance was set at a 2-sided *P* < .05, and analyses were conducted using R, version 4.0.4^34^ and RStudio, version 1.4.1106 (R Project for Statistical Computing).^36^

## Results

A total of 4360 patients were studied, including 256 (6%) with SR-TBI (mean [SD] age, 38.9 [18.1] years; 161 [63%] male; 1 [0.4%] Asian, 1 [0.4%] Black, 245 [96%] White, and 9 [4%] with unknown or missing race) and 4104 with NSR-TBI (mean [SD] age, 51.0 [20.2] years; 2773 [68%] male; 68 [2%] Asian, 61 [2%] Black, 3777 [92%] White, and 198 [5%] with unknown or missing race). Characteristics of the SR-TBI and NSR-TBI groups are given in Table 1 and the distribution of types of sports in Table 2. Patients with SR-TBI predominantly had mild injuries (162 of 247 [66%] had a GCS of 15) and were younger. The most common setting of SR-TBI was horseback riding (n = 57), followed by skiing (n = 44) and association football (soccer; n = 33).

**Table 2.** Characteristics of SR-TBI per Type of Sport

Sport	SR-TBI cases, No. (%) (n = 256)	Sex, No. (%)		Age, mean (SD), y	SR-TBI severity, No. (%)		
		Male	Female		Mild	Moderate	Severe
Team sports							
Association football	33 (13)	31 (94)	2 (6)	25 (9)	29 (88)	4 (12)	0
Rugby	8 (3)	5 (63)	3 (37)	23.5 (7)	8 (100)	0	0
Hockey (ice or field)	6 (2)	4 (67)	2 (33)	29 (10)	5 (83)	1 (17)	0
Other	17 (7)	10 (41)	7 (59)	26 (13)	16 (94)	1 (6)	0
Total	64	50 (78)	14 (22)	25 (10)	58 (91)	6 (9)	0
Individual sports							
Horseback riding	57 (22)	6 (11)	51 (89)	42.5 (17)	42 (74)	5 (9)	6 (11)
Skiing	44 (17)	36 (82)	8 (18)	43.5 (19)	30 (68)	4 (9)	9 (20)
Cycling^a^	23 (9)	18 (78)	5 (22)	45.5 (19)	21 (92)	0	2 (9)
Off-road vehicular sports	7 (3)	7 (100)	0	42.5 (27)	5 (71)	0	2 (29)
Rollerblading, scooter, skateboarding	5 (2)	4 (80)	1 (20)	35 (11)	5 (100)	0	0
Other	51 (20)	36 (71)	15 (29)	46 (19)	33 (65)	1 (2)	14 (27)
Total	187	107	80	43 (18)	136	10	37

Abbreviation: SR-TBI, sports-related traumatic brain injury.

^a^ In cycling, 37 cases were identified as sports or recreational, but 14 of them were excluded due to also being described as “road traffic accidents,” leaving 23 cases with SR-TBI caused by cycling. In the database, cyclists represented 563 (34.7%) of 1624 road traffic incidents.

### Factors Associated With SR-TBI

**Figure 1.**
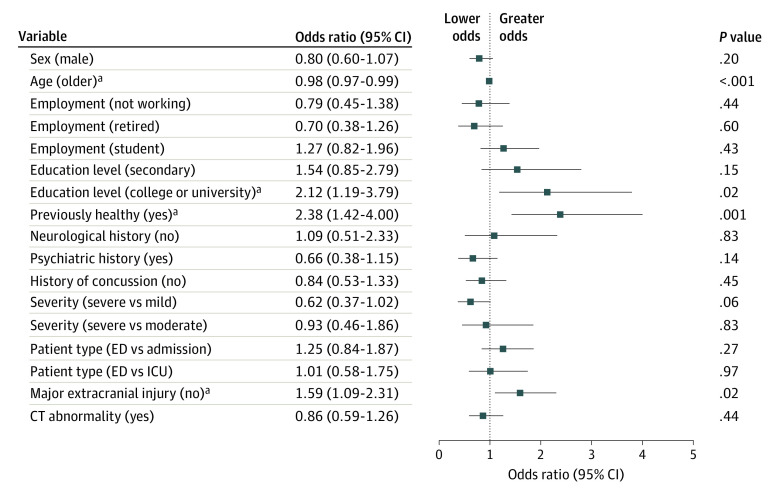
Multivariate Association of Background and Clinical Variables With Cause of Injury Odds ratios are given for membership of the sports-related traumatic brain injury group. Error bars indicate 95% CIs. CT indicates computed tomography; ED, emergency department; and ICU, intensive care unit. ^a^Significant at *P* < .05 level.

Odds ratios (ORs) for multivariate comparisons of background and clinical characteristics of SR-TBI and NSR-TBI are shown in Figure 1. Patients with SR-TBI were significantly younger (OR, 0.98; 95% CI, 0.97-0.99; *P* < .001); they were 2.12 (95% CI, 1.19-3.79) times more likely to have a university or college degree (*P* = .02) and were 2.38 (95% CI, 1.42-4.00) times more likely to be classified as healthy before their injury (*P* = .001). They were also 1.59 (95% CI, 1.09-2.31) times less likely to have a major extracranial injury (*P* = .02).

### Outcome Measures

**Figure 2.**
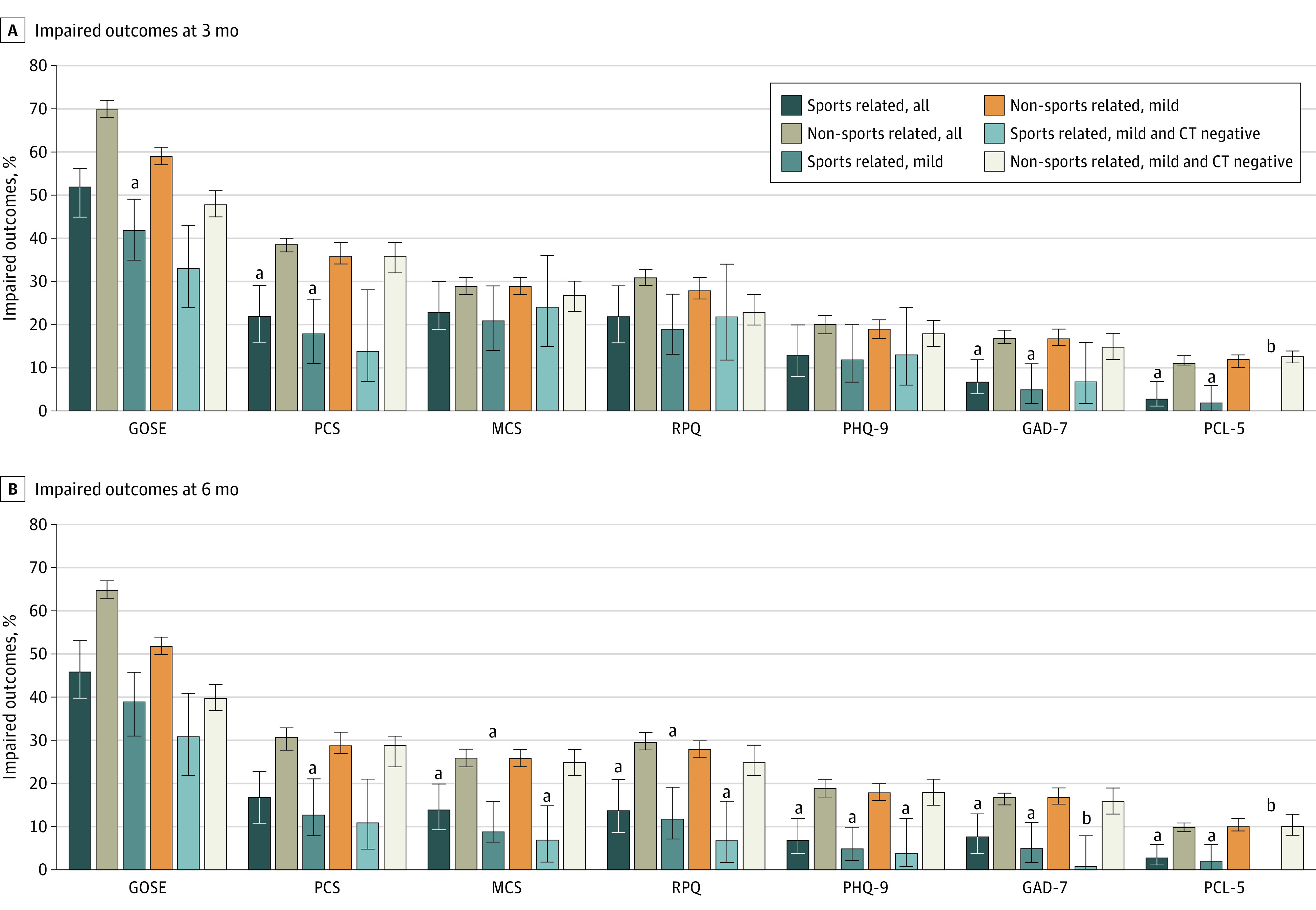
Percentages of Impaired Outcomes at 3 and 6 Months for Sports-Related and Non–Sports-Related Traumatic Brain Injury (TBI) in 3 Severity Groups (All Severities of Injury, Mild TBI, and Mild TBI With Negative Computed Tomography [CT] Results) GAD-7, Generalized Anxiety Disorder–7; GOSE, Glasgow Outcome Score–Extended; MCS, mental component summary; PCL-5, Posttraumatic Stress Disorder Checklist for *DSM-5*; PCS, physical component summary; PHQ-9, Patient Health Questionnaire–9; RPQ, Rivermead Post-concussion Symptoms Questionnaire. Error bars indicate 95 CIs. ^a^Significant at the *P* < .05 level after adjustment for covariates and false discovery rate correction. The bars are paired, and the comparison is between the sports-related TBI bar indicated and the corresponding non–sports-related TBI bar immediately to the right. ^b^Too few positive cases for analysis.

**Figure 3.**
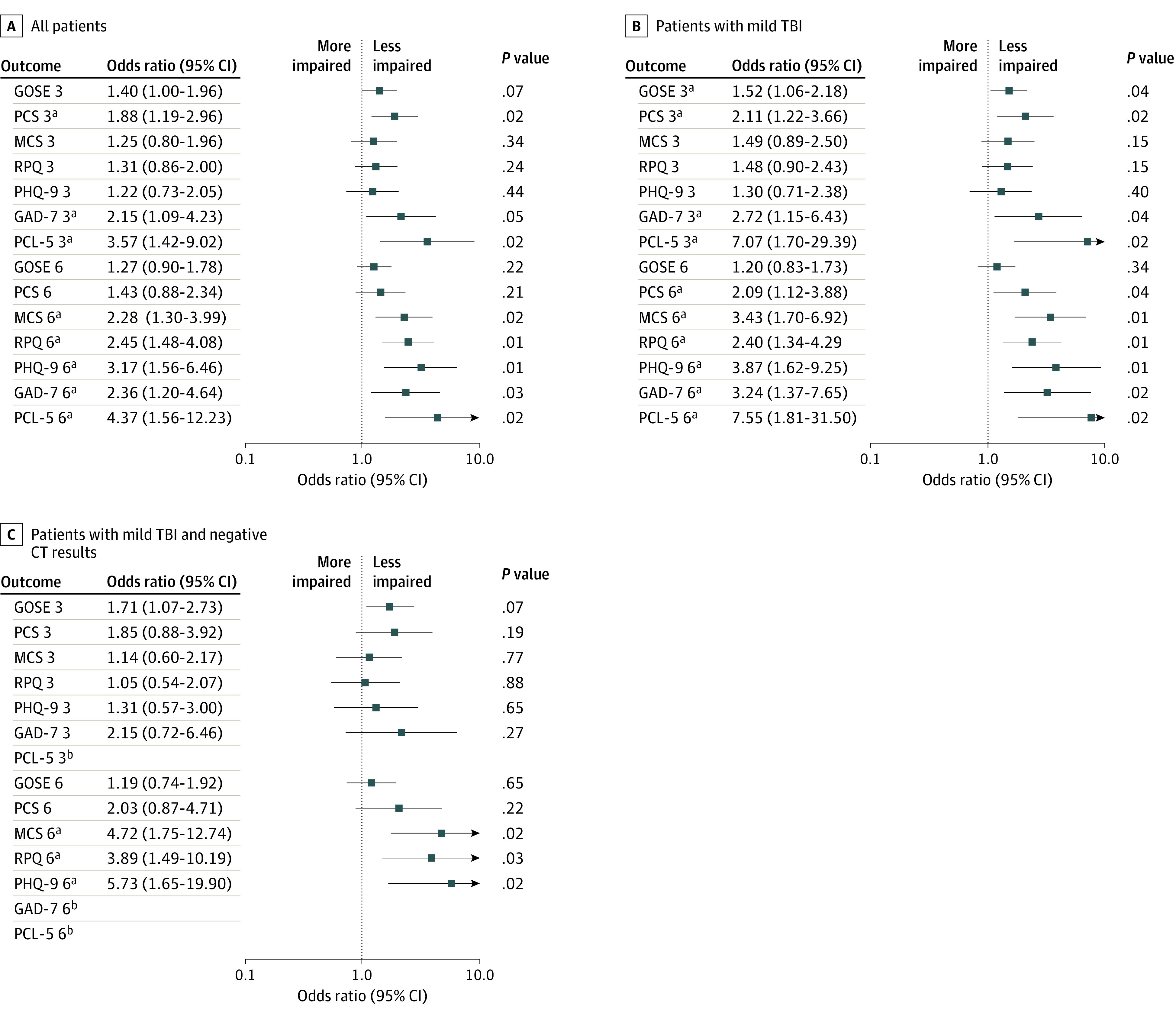
Odds Ratios for Comparison of Sports-Related Traumatic Brain Injury (TBI) vs Non–Sports-Related TBI on Outcomes at 3 and 6 Months, After Injury Adjusting for Covariates Larger odds ratios indicate better outcomes in the sports-related TBI group. *P* values are corrected for a false discovery rate of 5%. Error bars indicate 95% CIs. CT indicates computed tomography; GAD-7, Generalized Anxiety Disorder–7; GOSE, Glasgow Outcome Score–Extended; MCS, mental component summary; PCL-5, Posttraumatic Stress Disorder Checklist for *DSM-5*; PCS, physical component summary; PHQ-9, Patient Health Questionnaire–9; RPQ, Rivermead Post-concussion Symptoms Questionnaire. ^a^Significant at the *P* < .05 level after adjustment for covariates and false discovery rate correction. ^b^Too few positive cases for analysis.

Figure 2 summarizes our main findings as percentages of impairment in the SR-TBI and NSR-TBI groups, whereas Figure 3A, B, and C provides the results of adjusted comparisons using regression analyses. Completion rates for outcomes are provided in eTable 1 in Supplement 1 and percentages impaired on each assessment in eTable 2 in Supplement 1. The application of FDR correction to probabilities is detailed in eTable 3 in Supplement 1.

#### Three-Month Outcomes

The GOSE was the outcome with the greatest percentage of impairment in both the SR-TBI and NSR-TBI groups (Figure 2; eTable 2 in Supplement 1): 115 of 222 patients (52%) with outcomes in the SR-TBI group and 2390 of 3428 patients (70%) with in the NSR-TBI group had GOSE scores less than 8. The difference on the GOSE did not reach significance after correction (OR, 1.40; 95% CI, 1.00-1.96; *P* = .07). On the patient-reported outcomes, patients with SR-TBI were significantly less impaired than the NSR-TBI group on the PCS, GAD-7, and PCL-5 but not on the MCS, RPQ, and PHQ-9 (Figure 3A). In the SR-TBI group, impaired scores were relatively common on the PCS (36 [22%]), MCS (38 [23%]), and RPQ (35 [22%]), indicating that TBI symptoms and problems related to physical and mental health remained prominent at this time point, whereas clinically significant levels of depression (PHQ-9; 20 [13%]), anxiety (GAD-7; 11 [7%]), and PTSD (PCL-5; 5 [3%]) were relatively low.

Analysis of subgroups with milder injuries showed that a GOSE score less than 8 was present in 70 of 168 patients (42%) with mild SR-TBI and in 32 of 98 patients (33%) with mild SR-TBI and negative initial CT results compared with 1289 of 2202 (59%) and 524 of 1094 (48%), respectively, in the corresponding NSR-TBI groups. The difference between groups was significant for mild TBI (OR, 1.52; 95% CI, 1.06-2.18; *P* = .04) but not for mild TBI with negative CT results (OR, 1.71; 95% CI, 1.07-2.73; *P* = .07). Patients with mild SR-TBI were significantly less impaired than the NSR-TBI group on the PCS, GAD-7, and PCL-5 (Figure 3B), whereas there were no significant outcome differences between groups with mild SR-TBI and negative CT results (Figure 3C). Patterns of impaired scores in these less severely injured patients were similar to the group as a whole, and impaired scores were relatively common on the MCS (mild TBI: 17 [21%]; mild TBI with negative CT results: 17 [24%]) and RPQ (mild TBI: 23 [19%]; mild TBI with negative CT results: 14 [22%]).

#### Six-Month Outcomes

At the 6-month follow-up, the percentage of patients with GOSE scores less than 8 was still elevated: 103 of 223 patients (46%) with outcomes in the SR-TBI group had incomplete recovery (GOSE scores <8) compared with 2233 of 3451 patients (65%) in the NSR-TBI group (Figure 2). The difference between groups was not significant after adjustment (OR, 1.27; 95% CI, 0.90-1.78; *P* = .22). On the patient-reported outcomes, patients with SR-TBI were significantly less impaired than the NSR-TBI group on all assessments except the PCS (Figure 3A).

Analysis of subgroups with milder injuries showed that the number with GOSE scores less than 8 remained high at 6 months: 65 of 168 patients (39%) with mild SR-TBI and 30 of 98 (31%) with mild SR-TBI and negative CT results compared with 1146 of 2212 (52%) and 437 of 1095 (40%), respectively, in the corresponding NSR-TBI groups. The difference between groups on the GOSE was not significant for mild TBI (OR, 1.20; 95% CI, 0.83-1.73; *P* = .34) or mild TBI with negative CT results (OR, 1.19; 95% CI, 0.74-1.92; *P* = .65) (Figure 3B and C). However, patients with mild SR-TBI were significantly less impaired than the NSR-TBI group on all patient-reported outcomes (Figure 3B). Patients with mild SR-TBI and negative CT results were significantly less impaired on the MCS, RPQ, and PHQ-9, and low absolute numbers of impaired individuals precluded formal comparisons on the GAD-7 and PCL-5 (Figure 3C). Thus, although functional recovery on the GOSE remains incomplete at 6 months, TBI symptoms and problems related to mental health are consistently less frequent in the SR-TBI subgroups than the NSR-TBI subgroups.

#### Sensitivity Analysis

In a sensitivity analysis, we included all patients with outcome measures at both 3 and 6 months (eResults in Supplement 1). Findings for this analysis were similar to those using all cases, and the 95% CIs at 3 and 6 months overlapped for all measures (eFigure 2 in Supplement 1).

## Discussion

The findings of this cohort study confirm the presence of systematic background and clinical differences between groups with SR- and NSR-TBI. Patients with SR-TBI were younger, were more likely to be college students, and reported better health before injury than patients with NSR-TBI. In addition, SR-TBI was less likely to be accompanied by major extracranial injury. These differences are consistent with characteristics described previously.^6^ The current analysis demonstrates that differences are independently associated with SR-TBI and indicates that individuals with sports-related injuries represent a selective subsample of individuals with TBI.

After considering potential risk factors, the level of functional recovery on the GOSE in SR-TBI and NSR-TBI at 3 months was only significantly different in the subgroup with mild TBI, whereas at 6 months none of the comparisons remained significant. More than half (52%) of patients with SR-TBI had an incomplete recovery on the GOSE 3 months after the TBI, whereas nearly half (46%) were still not fully recovered at 6 months. Even in patients with an SR-TBI who incurred injuries that would be considered mild, this proportion was still high (39%) 6 months after injury. The finding that SR-TBI, including mild SR-TBI, was associated with persisting disability is consistent with recent findings from hospital-based studies of TBI, showing that injuries categorized as mild often have long-term consequences.^9^

At 3 months after injury, the SR-TBI group had fewer impaired outcomes on scales assessing anxiety and PTSD than those with NSR-TBI. On the other hand, impairment on the SF-12v2 MCS and the PHQ-9 was common in both groups. By 6 months, the SR-TBI group showed better recovery across all measures of mental health than the NSR-TBI group. The findings thus suggest that in the SR-TBI group, there is relative resistance to initial anxiety and stress and better long-term recovery from other mental health problems than in those with NSR-TBI. This finding is consistent with other reports, including, for example, the low observed prevalence of mental health problems in former professional soccer players.^37^ The proportion of patients with persistent postconcussion symptoms at 3 months is higher than generally described for college athletes,^38^ but the prevalence of symptoms diminishes by 6 months in parallel with the decrease in general mental health problems.

We found higher prevalence of impairment on the GOSE than the RPQ, which is consistent with a previous hospital-based study of mild TBI.^9^ Impairment on the RPQ was common at 3 months but had decreased substantially by 6 months. Historically, assessment of outcome after mild TBI, and specifically mild SR-TBI, has used scales such as the RPQ or similar symptom checklists. The presence of discrepancies among instruments indicates the desirability of a multidimensional approach to assessing outcomes after mild TBI to capture the full range of symptoms and problems that are present.

The CENTER-TBI cohort consists of patients who attended specialized neuroscience centers (equivalent to level I trauma centers in the US) who were triaged to receive CT. There are several hundred such specialized centers across Europe and the US; the findings are thus of direct relevance to a large group of patients but cannot be extrapolated to patients with SR-TBI who do not present at hospitals. The subgroup with GCS scores of 13 to 15 and negative CT findings are closest to having a sports concussion but nonetheless may well have sustained more significant injuries than a typical head injury sustained in sports. We used GCS scores of 13 to 15 to identify mild TBI because this definition is widely recognized in acute care and influences initial management. Other approaches, such as the American Congress of Rehabilitation Medicine criteria,^39^ would limit the number of cases considered to be mild. On the other hand, 80% of the SR-TBI sample had a GCS score of 13 to 15 and 66% had a GCS score of 15. Furthermore, advantages of this cohort study are its sampling technique, sample size, the range of sports activities represented, and the ability to control for confounding variables.

The outcome differences between groups suggest that there may be influences that were not controlled for in the analyses. For example, individuals who present to hospitals after sports injuries may have milder types of TBI that are not captured by the measures of severity used in this study. Athletes may receive superior postinjury support from sporting colleagues familiar with concussion. Light controlled exercise the first week after mild TBI can have positive effects on patients’ mental health and is beneficial for recovery.^40,41^ Patients with SR-TBI are presumably more likely to engage in exercise in the weeks after TBI than other patients due to their desire to return to sports. Individuals with SR-TBI have greater resilience to some of the consequences of TBI, in keeping with a benign view of the effects of concussion in sports. However, the presence of persisting limitations of functional outcome specifically warns against assuming an overoptimistic view of outcomes of mild SR-TBI. The fact that this group includes individuals who are relatively young and generally healthy before injury makes the presence of persisting problems particularly salient.

### Limitations

This study has some limitations. The study is based on patients triaged to CT attending specialist centers, and it is important to establish the extent of generalizability to other SR-TBI contexts. Furthermore, there was an absence of detailed records of preinjury problems and lack of information about treatment and support received after injury, which limits the explanations that can be offered for differences in outcome. Most cycling injuries recorded in the database were incurred in road traffic incidents, and we could not robustly identify those that involved a primary purpose for sports activity vs cycling as a mode of transport. As a result, most cycling injuries were considered transport related and included in the NSR-TBI group. A future study should explore this important cause of TBI. The CENTER-TBI study did not recruit an NSR-TBI control group, and although there are established cutoffs for outcomes, controls with extracranial injuries would potentially have allowed the specific influence of TBI to be better evaluated. Loss to follow-up is an issue and may bias findings toward a more pessimistic view of outcome, particularly in mild TBI.^9^ We addressed this problem for the GOSE using single imputation of missing values, but there were still gaps in the data. A number of important issues remain for future study. There is a need for work investigating the kinds of functional problems that individuals experience in the long term and the specific symptoms or domains that are driving differences in outcome. The effects of prior concussion and differences between types of sport and level of engagement (amateur vs professional) are also important issues for future research.

### Conclusions

In this cohort study, the sample with SR-TBI differed from the NSR-TBI group and was characterized by having lower risk factors for poor outcome. Six months after SR-TBI, recovery of mental health and postconcussion symptoms were better than in the NSR-TBI group, even after considering risk factors in the group. However, in contrast to the idea that recovery after mild TBI or concussion is unproblematic, we found that approximately one-third of individuals with SR-TBI with GCS scores of 13 to 15 and negative CT results had persisting disability at 6 months. The findings have implications for injury prevention and management and indicate that even among individuals with sports injuries considered to be mild, many would benefit from systematic follow-up.

## Notes

Supplement 1. **eFigure 1.** CONSORT Diagram **eTable 1.** Completion Rates for Outcomes **eTable 2.** Numbers of Impaired Outcomes **eTable 3.** Correction for Multiple Comparisons for All TBI Patients, mTBI Patients, and Patients With mTBI and Negative CT Scan on Admission **eResults.** Supplementary Analysis **eFigure 2.** SR-TBI Outcomes Odds Ratio at 3- and 6-Months Post-Injury for Patients With Assessments Both at 3- and 6-Months Post-Injury

Supplement 2.
Nonauthor Collaborators

Supplement 3.
Data Sharing Statement
