# Supplementary material for: Contrasting Characteristics and Outcomes of Sports-Related and Non–Sports-Related Traumatic Brain Injury
Source: JAMA Netw Open. 2024 Jan 24;7(1):e2353318. doi: 10.1001/jamanetworkopen.2023.53318 (PMC10809021; doi:10.1001/jamanetworkopen.2023.53318)
Supplement: Supplement 1. — eFigure 1. CONSORT Diagram eTable 1. Completion Rates for Outcomes eTable 2. Numbers of Impaired Outcomes eTable 3. Correction for Multiple Comparisons for All TBI Patients, mTBI Patients, and Patients With mTBI and Negative CT Scan on Admission eResults. Supplementary Analysis eFigure 2. SR-TBI Outcomes Odds Ratio at 3- and 6-Months Post-Injury for Patients With Assessments Both at 3- and 6-Months Post-Injury [file jamanetwopen-e2353318-s001.pdf]

Supplementary Online Content

Ntikas M, Stewart W, Ietswaart M, et al; CENTER-TBI participants and investigators. Contrasting characteristics and outcomes of sports- and non–sports-related traumatic brain injury. *JAMA Netw Open*. 2024;7(1):e2353318. doi:10.1001/jamanetworkopen.2023.53318

eFigure 1. CONSORT Diagram
eTable 1. Completion Rates for Outcomes
eTable 2. Numbers of Impaired Outcomes
eTable 3. Correction for Multiple Comparisons for All TBI Patients, mTBI Patients, and Patients With mTBI and Negative CT Scan on Admission
eResults. Supplementary Analysis
eFigure 2. SR-TBI Outcomes Odds Ratio at 3- and 6-Months Post-Injury for Patients With Assessments Both at 3- and 6-Months Post-Injury

This supplementary material has been provided by the authors to give readers additional information about their work.

© 2024 Ntikas M et al. *JAMA Network Open.*

eFigure 1. CONSORT flow diagram.

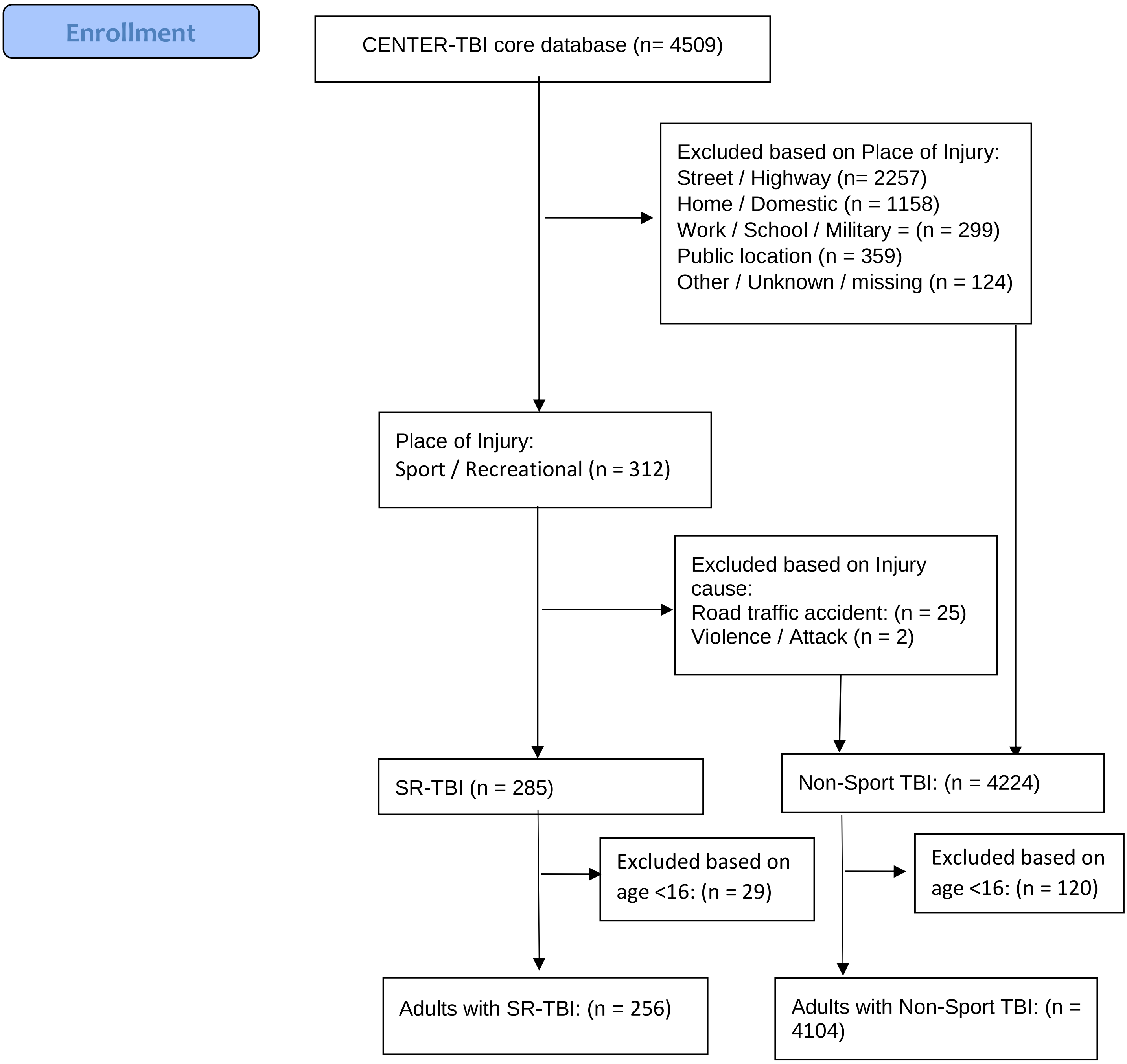

© 2024 Ntikas M et al. *JAMA Network Open.*

eTable 1a. Completion rates for outcomes at each timepoint, % out of 4360 patients

Outcome	N at 3 months (%)	N at 6 months (%)
GOSE	3650 (84)	3674 (84)
SF-12v2 PCS	2222 (51)	2224 (51)
SF-12v2 MCS	2222 (51)	2224 (51)
RPQ	2084 (48)	2167 (50)
PHQ-9	2044 (47)	2125 (49)
GAD-7	2039 (47)	2122 (49)
PCL-5	2037 (47)	2116 (49)

Note. N = sample size.

eTable 1b Completion rates for outcomes at each timepoint, % out of 256 SR-TBI patients

Outcome (SR-TBI)	N at 3 months (%)	N at 6 months (%)
GOSE	222 (87)	223 (87)
SF-12v2 PCS	165 (64)	160 (63)
SF-12v2 MCS	165 (64)	160 (63)
RPQ	158 (62)	156 (61)
PHQ-9	152 (59)	155 (60)
GAD-7	151 (59)	153 (60)
PCL-5	157 (61)	155 (60)

Note. N = sample size.

eTable 1c Completion rates for outcomes at each timepoint, % out of 4104 non-SR-TBI patients

Outcome (TBI)	N at 3 months (%)	N at 6 months (%)
GOSE	3428 (84)	3451 (84)
SF-12v2 PCS	2057 (50)	2064 (50)
SF-12v2 MCS	2057 (50)	2064 (50)
RPQ	1926 (47)	2011 (49)
PHQ-9	1892 (46)	1970 (48)
GAD-7	1888 (46)	1969 (48)
PCL-5	1880 (46)	1961 (48)

Note. N = sample size.

© 2024 Ntikas M et al. *JAMA Network Open.*

eTable 2. Numbers (percentages and 95% confidence intervals) of impaired outcomes at 3 and 6 months.

Timepoint	Outcome measure	SR-TBI<br>n (% [95% CI])	mild SR-TBI<br>n (% [95% CI])	mild SR-TBI & negative CT<br>n (% [95% CI])	Non-sport TBI<br>n (% [95% CI])	Non-sport mild TBI<br>n (% [95% CI])	Non-sport mild TBI & negative CT<br>n (% [95% CI])
3 months	GOSE	115 (52 [45-56])	70 (42 [35-49])	32 (33 [24-43])	2390 (70 [68-72])	1289 (59 [57-61])	524 (48 [45-51])
	RPQ	35 (22 [16-29])	23 (19 [13-27])	14 (22 [12-34])	587 (31 [29-33])	409 (28 [26-31])	167 (23 [20-27])
	PHQ-9	20 (13 [8-20])	14 (12 [7-20])	8 (13 [6-24])	379 (20 [18-22])	270 (19 [17-21])	126 (18 [15-21])
	GAD-7	11 (7 [4-12])	6 (5 [2-11])	4 (7 [2-16])	325 (17 [16-19])	238 (17 [15-19])	108 (15 [12-18])
	PCL-5	5 (3 [1-7])	2 (2 [0-6])	0	213 (11 [10-13])	165 (12 [10-13])	83 (12 [10-14])
	PCS	36 (22 [16-29])	22 (18 [11-26])	10 (14 [7 – 28])	787 (38 [36-40])	511 (36 [34-39])	247 (36 [32-39])
	MCS	38 (23 [19-30])	27 (21 [14-29])	17 (24 [15-36])	580 (29 [27-31])	405 (29 [27-31])	181 (27 [23-30])
6 months	GOSE	103 (46 [40-53])	65 (39 [31-46])	30 (31 [22-41])	2233 (65 [63-67])	1146 (52 [50-54])	437 (40 [37-43])
	RPQ	22 (14 [9-21])	15 (12 [7-19])	5 (7 [2-16])	603 (30 [28-32])	411 (28 [26-30])	184 (25 [22-29])
	PHQ-9	11 (7 [4-12])	6 (5 [2-10])	3 (4 [1-12])	370 (19 [17-21])	257 (18 [16-20])	127 (18 [15-21])
	GAD-7	12 (8 [4-13])	6 (5 [2-11])	1 (1 [0-8])	328 (17 [15-18])	244 (17 [15-19])	110 (16 [13-19])
	PCL-5	4 (3 [1-6])	2 (2 [0-6])	0	196 (10 [9-11])	151 (10 [9-12])	74 (10 [8-13])
	PCS	27 (17 [11-23])	17 (13 [8-21])	7 (11 [5-21])	631 (31 [28-33])	410 (29 [27-32])	189 (29 [24-31])
	MCS	22 (14 [9-20])	11 (9 [5-16])	5 (7 [2-15])	524 (26 [24-28])	354 (26 [24-28])	166 (25 [22-28])

Abbreviations: GOSE = Glasgow Outcome Score Extended. RPQ = Rivermead Post-concussion Symptoms Questionnaire. PHQ-9 = Patient Health Questionnaire 9. GAD-7 = Generalised Anxiety Disorder 7. PCL-5 = Posttraumatic Stress Disorder Checklist 5. PCS = Physical Component Summary. MCS = Mental Component Summary.

© 2024 Ntikas M et al. *JAMA Network Open.*

eTable 3a. Correction for multiple comparisons with the use of FDR (false discovery rate) for the comparison of the outcomes of SR-TBI and non-sports TBI for all TBI patients. The table gives the original *P* values from binary logistic regression, the critical *P* values at a 5% FDR rate, and the FDR corrected *P* values.

Rank	Variable	Original *P* value	Critical Value	Adjusted *P* value	Significance
1	RPQ 6	0.001	0.004	0.01	Yes
2	PHQ-9 6	0.002	0.007	0.01	Yes
3	PCL-5 6	0.004	0.01	0.02	Yes
4	PCL-5 3	0.005	0.01	0.02	Yes
5	MCS 6	0.007	0.02	0.02	Yes
6	PCS 3	0.007	0.02	0.02	Yes
7	GAD-7 6	0.01	0.03	0.03	Yes
8	GAD-7 3	0.03	0.03	0.05	Yes
9	GOSE 3	0.05	0.03	0.07	No
10	PCS 6	0.15	0.04	0.21	No
11	GOSE 6	0.17	0.04	0.22	No
12	RPQ 3	0.21	0.04	0.24	No
13	MCS 3	0.32	0.05	0.34	No
14	PHQ-9 3	0.44	0.05	0.44	No

© 2024 Ntikas M et al. *JAMA Network Open.*

eTable 3b. Correction for multiple comparisons with the use of FDR (false discovery rate) for the comparison of the outcomes of SR-TBI and non-sports TBI for patients with mTBI. The table gives the original *P* values from binary logistic regression, the critical *P* values at a 5% FDR rate, and the FDR corrected *P* values.

Rank	Variable	Original *P* value	Critical Value	Adjusted *P* value	Significance
1	MCS 6	<.001	0.004	0.008	Yes
2	PHQ-9 6	0.002	0.007	0.01	Yes
3	RPQ 6	0.003	0.01	0.01	Yes
4	PCL-5 6	0.005	0.01	0.02	Yes
5	GAD-7 6	0.007	0.02	0.02	Yes
6	PCL-5 3	0.007	0.02	0.02	Yes
7	PCS 3	0.008	0.03	0.02	Yes
8	GAD-7 3	0.02	0.03	0.04	Yes
9	GOSE 3	0.02	0.03	0.04	Yes
10	PCS 6	0.03	0.04	0.04	Yes
11	RPQ 3	0.12	0.04	0.15	No
12	MCS 3	0.13	0.04	0.15	No
13	GOSE 6	0.32	0.05	0.34	No
14	PHQ-9 3	0.4	0.05	0.4	No

Note. GOSE = Glasgow Outcome Score Extended. RPQ = Rivermead Post-concussion Symptoms Questionnaire. PHQ-9 = Patient Health Questionnaire. GAD-7 = Generalised Anxiety Disorder. PCL-5 = Posttraumatic Stress Disorder Checklist. PCS = Physical Component Summary. MCS = Mental Component Summary.

© 2024 Ntikas M et al. *JAMA Network Open.*

eTable 3c. Correction for multiple comparisons with the use of FDR (false discovery rate) for the comparison of outcomes of SR-TBI and non-sports TBI for patients with mTBI and negative CT scan on admission. The table gives the original *P* values from binary logistic regression, the critical *P* values at a 5% FDR rate, and the FDR corrected *P* values.

Rank	Variable	Original *P* value	Critical Value	Adjusted *P* value	Significance
1	MCS 6	0.002	0.005	0.02	Yes
2	RPQ 6	0.006	0.009	0.03	Yes
3	PHQ-9 6	0.006	0.01	0.02	Yes
4	GOSE 3	0.03	0.02	0.07	No
5	PCS 6	0.1	0.02	0.22	No
6	PCS 3	0.1	0.03	0.19	No
7	GAD-7 3	0.17	0.03	0.27	No
8	GOSE 6	0.48	0.04	0.65	No
9	PHQ-9 3	0.53	0.04	0.65	No
10	MCS 3	0.7	0.05	0.77	No
11	RPQ 3	0.88	0.05	0.88	No

Note. GOSE = Glasgow Outcome Score Extended. RPQ = Rivermead Post-concussion Symptoms Questionnaire. PHQ-9 = Patient Health Questionnaire. GAD-7 = Generalised Anxiety Disorder. PCL-5 = Posttraumatic Stress Disorder Checklist. PCS = Physical Component Summary. MCS = Mental Component Summary.

© 2024 Ntikas M et al. *JAMA Network Open.*

eResults. Supplementary Analysis

To investigate outcome trends from 3 to 6 months an analysis was conducted of data from patients assessed at both timepoints. The number of patients with outcome information both at 3 and 6 months can be found on Table 2 of the supplementary material. Binary logistic regressions, similar to the ones performed in whole database, were done on those samples.

SR-TBI patients were found to be 1.4[1.003-1.96] times more likely to have returned to normal life at 3 months (*P* = .05) compared to patient with TBI from other causes, this difference was absent at 6 months post-injury (*P* = .18; Figure 2). SR-TBI patients were also found to be 2.27 [1.35-3.83] times more likely to have better physical health related quality of life 3 months post-injury (*P* = .002), but this difference was again absent at 6 months (*P* = .23). Contrary to that, SR-TBI patients not more likely to have better MCS score 3 months (*P* = .172) post injury but were 2.23 [1.19-4.16] times more likely to have a better MCS score at 6 months (*p* = .01).

SR-TBI patients concussion symptom outcomes did not differ from the rest of the sample at the 3 months timepoint (*P* = .15), however at 6 months SR-TBI patients were 2.46[1.39-4.38] times less likely to have persistent post-concussion symptoms (*P*=.002).

Concerning mental health outcomes, SR-TBI patients did not differ in depression symptoms at 3 months post injury (*P* = .45) but were 3.89[1.54-9.83] times less likely to have depressive symptoms compared to the rest of the sample (*P* = .005). SR-TBI patients were found to be 2.65 [1.19-5.89] and 3.29 [1.30-8.36] times less likely to have anxiety symptoms at 3 and 6 months post-injury respectively. Similarly, SR-TBI patients were found to be 3.49[1.25-9.76] and 6.35[1.53-26.31] times less likely to have PTSD symptoms at 3 and 6 months post-injury respectively.

All *P* values remained below the chosen level of significance (.05) after correction for multiple comparison, apart from the GOSE score at 3 months.

The number of patients with mTBI and mTBI with negative CT scan with outcome measures both at 3 and 6 months was too small, so analyses on those subsamples were not performed.

© 2024 Ntikas M et al. *JAMA Network Open.*

eFigure 2. SR-TBI outcomes odds ratio at 3- and 6-months post-injury (Only for patients with assessments both at 3- and 6-months post-injury).

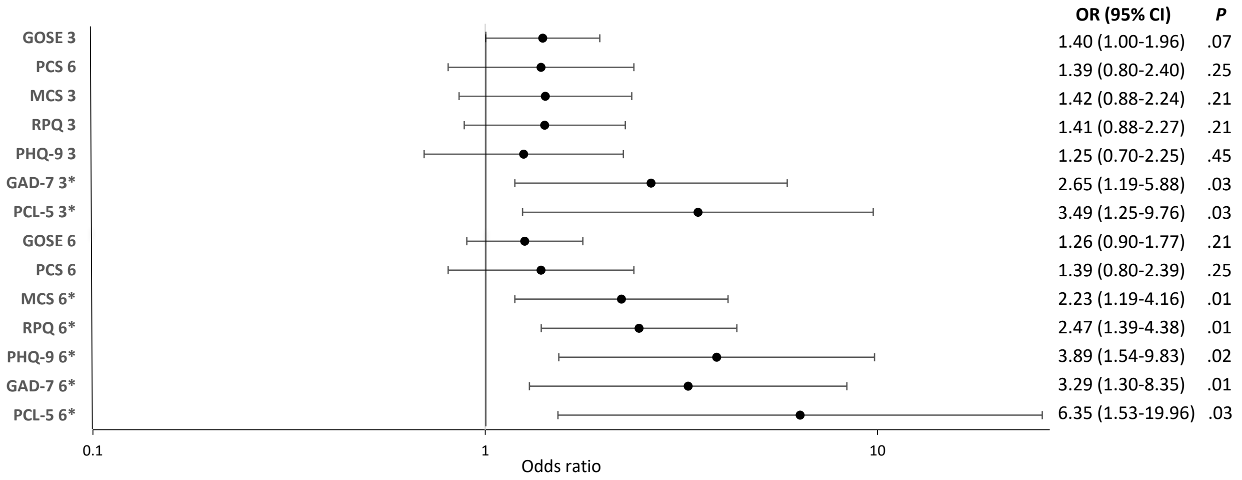

Note. *Denotes significant *P* value. GOSE = Glasgow Outcome Score Extended. RPQ = Rivermead Post-concussion Symptoms Questionnaire. PHQ-9 = Patient Health Questionnaire. GAD-7 = Generalised Anxiety Disorder. PCL-5 = Posttraumatic Stress Disorder Checklist. PCS = Physical Component Summary. MCS = Mental Component Summary. Horizontal lines represent 95% confidence intervals. Vertical like represents an odds ratio of 1.

© 2024 Ntikas M et al. *JAMA Network Open.*
