# Supplementary material for: Contrasting Characteristics and Outcomes of Sports-Related and Non–Sports-Related Traumatic Brain Injury
Source: JAMA Netw Open. 2024 Jan 24;7(1):e2353318. doi: 10.1001/jamanetworkopen.2023.53318 (PMC10809021; doi:10.1001/jamanetworkopen.2023.53318)
Supplement: Supplement 2. — Nonauthor Collaborators [file jamanetwopen-e2353318-s002.pdf]

*Indicates required information. Only first name, last name, and suffix will appear in PubMed.

*Group Name(s): The CENTER-TBI participants and investigators							
***First Name and Middle Initial(s)**	***Last Name**	***Suffix (eg, Jr, III)**	Academic Degrees	Institution	Location (city, state/province, country)	Role or Contribution, eg, chair, principal investigator	Group (if more than 1 Group listed in the byline) and/or Subgroup (eg, Steering Committee)
Cecilia	Ackerlund			Karolinska Institutet, INCF Internatior	Stockholm, Sweden	CENTER-TBI Participant + Investigator	
Krisztina	Amrein			János Szentágothai Research Centre,	Pécs, Hungary	CENTER-TBI Participant + Investigator	
Nada	Andelic			Division of Surgery and Clinical Neurc	Oslo, Norway	CENTER-TBI Participant + Investigator	
Lasse	Andreassen			Department of Neurosurgery, Univer:	Tromso, Norway	CENTER-TBI Participant + Investigator	
Audny	Anke			Department of Physical Medicine anc	Tromso, Norway	CENTER-TBI Participant + Investigator	
Anna	Antoni			Trauma Surgery, Medical University V	Vienna, Austria	CENTER-TBI Investigator	
Gérard	Audibert			Department of Anesthesiology & Inte	Nancy, France	CENTER-TBI Investigator	
Philippe	Azouvi			Raymond Poincare hospital, Assistan	Paris, France	CENTER-TBI Participant	
Maria Luisa	Azzolini			Department of Anesthesiology & Inte	Milan, Italy	CENTER-TBI Investigator	
Ronald	Bartels			Department of Neurosurgery, Radbo	Nijmegen, Netherlands	CENTER-TBI Investigator	
Pál	Barzó			Department of Neurosurgery, Univer:	Szeged, Hungary	CENTER-TBI Investigator	
Romuald	Beauvais			International Projects Management, ,	Munchen, Germany	CENTER-TBI Participant	
Ronny	Beer			Department of Neurology, Neurologi	Innsbruck, Austria	CENTER-TBI Investigator	
Bo-Michael	Bellander			Deparment of Neurosurgery & Anest	Stockholm, Sweden	CENTER-TBI Investigator	
Antonio	Belli			NIHR Surgical Reconstruction and Mi	Birmingham, UK	CENTER-TBI Investigator	
Habib	Benali			Anesthesie-Réanimation, Assistance I	Paris, France	CENTER-TBI Participant + Investigator	
Maurizio	Berardino			Department of Anesthesia & ICU, AO	Torino, Italy	CENTER-TBI Investigator	
Luigi	Beretta			Department of Anesthesiology & Inte	Milan, Italy	CENTER-TBI Investigator	
Morten	Blaabjerg			Department of Neurology, Odense U	Odense, Denmark	CENTER-TBI Participant + Investigator	
Peter	Bragge			BehaviourWorks Australia, Monash S	Melbourne, Australia	CENTER-TBI Participant	
Alexandra	Brazinova			Department of Public Health, Faculty	Trnava, Slovakia	CENTER-TBI Participant	
Vibeke	Brinck			Quesgen Systems Inc., Burlingame, C	Burlingame, California, USA	CENTER-TBI Participant	
Joanne	Brooker			Australian & New Zealand Intensive (	Melbourne, Australia	CENTER-TBI Participant	
Camilla	Brorsson			Department of Surgery and Periopera	Umea, Sweden	CENTER-TBI Investigator	
Andras	Buki			Department of Neurosurgery, Medica	Pecs, Hungary	CENTER-TBI Participant + Investigator	
Monika	Bullinger			Department of Medical Psychology, U	Hamburg, Germany	CENTER-TBI Associated Participant	
Manuel	Cabeleira			Brain Physics Lab, Division of Neuros	Cambridge, UK	CENTER-TBI Participant + Investigator	
Alessio	Caccioppola			Neuro ICU, Fondazione IRCCS Cà G	Milan, Italy	CENTER-TBI Participant + Investigator	
Emiliana	Calappi			Neuro ICU, Fondazione IRCCS Cà G	Milan, Italy	CENTER-TBI Participant + Investigator	

*Indicates required information. Only first name, last name, and suffix will appear in PubMed.

***First Name and Middle Initial(s)**	***Last Name**	***Suffix (eg, Jr, III)**	Academic Degrees	Institution	Location (city, state/province, country)	Role or Contribution, eg, chair, principal investigator	Group (if more than 1 Group listed in the byline) and/or Subgroup (eg, Steering Committee)
Maria Rosa	Calvi			Department of Anesthesiology & Inte	Milan, Italy	CENTER-TBI Investigator	
Peter	Cameron			ANZIC Research Centre, Monash Univ	Melbourne, Australia	CENTER-TBI Participant	
Guillermo	Carbayo Lozano			Department of Neurosurgery, Hospit	Bilbao, Spain	CENTER-TBI Investigator	
Marco	Carbonara			Neuro ICU, Fondazione IRCCS Cà G	Milan, Italy	CENTER-TBI Participant + Investigator	
Ana M.	Castaño-León			Department of Computing, Imperial (	London, UK	CENTER-TBI Participant	
Simona	Cavallo			Department of Anesthesia & ICU, AO	Torino, Italy	CENTER-TBI Investigator	
Giorgio	Chevallard			NeuroIntensive Care, Niguarda Ho	Niguarda, Italy	CENTER-TBI Investigator	
Arturo	Chieregato			NeuroIntensive Care, Niguarda Ho	Niguarda, Italy	CENTER-TBI Investigator	
Giuseppe	Citerio			School of Medicine and Surgery, Univ	Milano, Italy	CENTER-TBI Participant + Investigator + MC	
Hans	Clusmann			Department of Neurosurgery, Medica	Aachen, Germany	CENTER-TBI Investigator	
Mark Steven	Coburn			Department of Anesthesiology an	Bonn, Germany	CENTER-TBI Investigator	
Jonathan	Coles			Department of Anesthesia & Neuroin	Cambridge, UK	CENTER-TBI Participant + Investigator	
Jamie D.	Cooper			School of Public Health & PM, Monas	Melbourne, Australia	CENTER-TBI Participant + Investigator	
Marta	Correia			Radiology/MRI department, MRC Cog	Cambridge, UK	CENTER-TBI Associated Participant	
Amra	Čović			Institute of Medical Psychology and M	Göttingen, Germany	CENTER-TBI Participant	
Nicola	Curry			Oxford University Hospitals NHS Trus	Oxford, UK	CENTER-TBI Participant	
Endre	Czeiter			Department of Neurosurgery, Medica	Pecs, Hungary	CENTER-TBI Participant + Investigator	
Marek	Czosnyka			Brain Physics Lab, Division of Neurosu	Cambridge, UK	CENTER-TBI Participant + Investigator	
Claire	Dahyot-Fizelier			Intensive care Unit, CHU Poitiers, Poi	Poitiers, France	CENTER-TBI Investigator	
Paul	Dark			University of Manchester NIHR Biome	Salford, UK	CENTER-TBI Investigator	
Helen	Dawes			Movement Science Group, Faculty of	Oxford, UK	CENTER-TBI Participant	
Véronique	De Keyser			Department of Neurosurgery, Antwe	Edegem, Belgium	CENTER-TBI Participant + Investigator	
Vincent	Degos			Anesthesie-Réanimation, Assistance I	Paris, France	CENTER-TBI Participant + Investigator	
Francesco	Della Corte			Department of Anesthesia & Intensiv	Novara, Italy	CENTER-TBI Investigator	
Hugo	den Boogert			Department of Neurosurgery, Radbo	Nijmegen, Netherlands	CENTER-TBI Investigator	
Bart	Depreitere			Department of Neurosurgery, Univer	Leuven, Belgium	CENTER-TBI Investigator	
Đula	Đilvesi			Department of Neurosurgery, Clinical	Novi Sad, Serbia	CENTER-TBI Investigator	
Abhishek	Dixit			Division of Anaesthesia, University of	Cambridge, UK	CENTER-TBI Participant + Investigator	
Emma	Donoghue			ANZIC Research Centre, Monash Univ	Melbourne, Australia	CENTER-TBI Participant	
Jens	Dreier			Interdisciplinary Neuro Intensive Care	Berlin, Germany	CENTER-TBI Participant + Investigator	
Guy-Loup	Dulière			Intensive Care Unit, CHR Citadelle , Li	Liège, Belgium	CENTER-TBI Investigator	

*Indicates required information. Only first name, last name, and suffix will appear in PubMed.

*First Name and Middle Initial(s)	*Last Name	*Suffix (eg, Jr, III)	Academic Degrees	Institution	Location (city, state/province, country)	Role or Contribution, eg, chair, principal investigator	Group (if more than 1 Group listed in the byline) and/or Subgroup (eg, Steering Committee)
Ari	Ercole			Division of Anaesthesia, University of	Cambridge, UK	CENTER-TBI Participant + Investigator	
Patrick	Esser			Movement Science Group, Faculty of	Oxford, UK	CENTER-TBI Participant	
Erzsébet	Ezer			Department of Anaesthesiology and I	Pécs, Hungary	CENTER-TBI Participant + Investigator	
Martin	Fabricius			Departments of Neurology, Clinical N	Copenhagen, Denmark	CENTER-TBI Participant + Investigator	
Valery L.	Feigin			National Institute for Stroke and App	Auckland, New Zealand	CENTER-TBI Participant	
Kelly	Foks			Department of Neurology, Erasmus M	Rotterdam, Netherlands	CENTER-TBI Participant + Investigator	
Shirin	Frisvold			Department of Anesthesiology and Ir	Tromso, Norway	CENTER-TBI Investigator	
Alex	Furmanov			Department of Neurosurgery, Hadass	Jerusalem, Israel	CENTER-TBI Investigator	
Pablo	Gagliardo			Fundación Instituto Valenciano de Ne	Valencia, Spain	CENTER-TBI Associated Participant	
Damien	Galanaud			Anesthesie-Réanimation, Assistance I	Paris, France	CENTER-TBI Participant + Investigator	
Dashiell	Gantner			ANZIC Research Centre, Monash Univ	Melbourne, Australia	Oz ENTER	
Guoyi	Gao			Department of Neurosurgery, Shangh	Shanghai, China	CENTER-TBI Participant	
Pradeep	George			Karolinska Institutet, INCF Internatior	Stockholm, Sweden	CENTER-TBI Participant	
Alexandre	Ghuysen			Emergency Department, CHU , Liège,	Liège, Belgium	CENTER-TBI Investigator	
Lelde	Giga			Neurosurgery clinic, Pauls Stradins Cl	Riga, Latvia	CENTER-TBI Investigator	
Ben	Glocker			Department of Computing, Imperial C	London, UK	CENTER-TBI Participant	
Jagoš	Golubović			Department of Neurosurgery, Clinical	Novi Sad, Serbia	CENTER-TBI Investigator	
Pedro A.	Gomez			Department of Neurosurgery, Hospita	Madrid, Spain	CENTER-TBI Investigator	
Johannes	Gratz			Department of Anesthesia, Critical Ca	Vienna, Austria	CENTER-TBI Participant	
Benjamin	Gravesteijn			Department of Public Health, Erasmu	Rotterdam, Netherlands	CENTER-TBI Participant	
Francesca	Grossi			Department of Anesthesia & Intensiv	Novara, Italy	CENTER-TBI Investigator	
Russell L.	Gruen			College of Health and Medicine, Aust	Canberra, Australia	CENTER-TBI Associated Participant	
Deepak	Gupta			Department of Neurosurgery, Neuros	Delhi, India	CENTER-TBI Associated participant + Investigator	
Juanita A.	Haagsma			Department of Public Health, Erasmu	Rotterdam, Netherlands	CENTER-TBI Participant	
Iain	Haitsma			Department of Neurosurgery, Erasmu	Rotterdam, Netherlands	CENTER-TBI Investigator	
Raimund	Helbok			Department of Neurology, Neurologi	Innsbruck, Austria	CENTER-TBI Investigator	
Eirik	Helseth			Department of Neurosurgery, Oslo U	Oslo, Norway	CENTER-TBI Participant + Investigator	
Lindsay	Horton			Division of Psychology, University of S	Stirling, UK	CENTER-TBI Participant	
Jilske	Huijben			Department of Public Health, Erasmu	Rotterdam, Netherlands	CENTER-TBI Participant	
Peter J.	Hutchinson			Division of Neurosurgery, Departmen	Cambridge, UK	CENTER-TBI Participant + Investigator	
Bram	Jacobs			Department of Neurology, University	Groningen, Netherlands	CENTER-TBI Investigator	

Supplemental Online Content: Nonauthor Collaborators

*Indicates required information. Only first name, last name, and suffix will appear in PubMed.

*First Name and Middle Initial(s)	*Last Name	*Suffix (eg, Jr, III)	Academic Degrees	Institution	Location (city, state/province, country)	Role or Contribution, eg, chair, principal investigator	Group (if more than 1 Group listed in the byline) and/or Subgroup (eg, Steering Committee)
Stefan	Jankowski			Neurointensive Care , Sheffield Teach	Sheffield, UK	CENTER-TBI Investigator	
Mike	Jarrett			Quesgen Systems Inc., Burlingame, C	Burlingame, California, USA	CENTER-TBI Participant	
Ji-yao	Jiang			Department of Neurosurgery, Shangh	Shanghai, China	CENTER-TBI Participant	
Faye	Johnson			Salford Royal Hospital NHS Foundatio	Salford, UK	CENTER-TBI Investigator	
Kelly	Jones			National Institute for Stroke and App	Auckland, New Zealand	CENTER-TBI Participant	
Mladen	Karan			Department of Neurosurgery, Clinical	Novi Sad, Serbia	CENTER-TBI Investigator	
Angelos G.	Kolias			Division of Neurosurgery, Departmen	Cambridge, UK	CENTER-TBI Participant + Investigator	
Erwin	Kompanje			Department of Intensive Care and De	Rotterdam, Netherlands	CENTER-TBI Participant	
Daniel	Kondziella			Departments of Neurology, Clinical N	Copenhagen, Denmark	CENTER-TBI Investigator	
Evgenios	Kornaropoulos			Division of Anaesthesia, University	Cambridge, UK	CENTER-TBI Investigator	
Lars-Owe	Koskinen			Department of Neurosurgery, Umea	Umea, Sweden	CENTER-TBI Investigator	
Noémi	Kovács			Hungarian Brain Research Program -	Pécs, Hungary	CENTER-TBI Participant + Investigator	
Ana	Kowark			Department of Anesthesiology, Unive	Aachen, Germany	CENTER-TBI Investigator	
Alfonso	Lagares			Department of Neurosurgery, Hospita	Madrid, Spain	CENTER-TBI Investigator	
Linda	Lanyon			Karolinska Institutet, INCF Internation	Stockholm, Sweden	CENTER-TBI Participant + MC	
Steven	Laureys			Cyclotron Research Center , Universit	Liège, Belgium	CENTER-TBI Participant + Investigator	
Fiona	Lecky			Centre for Urgent and Emergency	Sheffield, UK	CENTER-TBI Participant + Investigator + MC	
Didier	Ledoux			Cyclotron Research Center , Universit	Liège, Belgium	CENTER-TBI Participant + Investigator	
Rolf	Lefering			Institute of Research in Operative Me	Cologne, Germany	CENTER-TBI Participant	
Valerie	Legrand			VP Global Project Management CNS,	Paris, France	CENTER-TBI Participant	
Aurelie	Lejeune			Department of Anesthesiology-Intens	Lille, France	CENTER-TBI Investigator	
Leon	Levi			Department of Neurosurgery, Ramba	Haifa, Israel	CENTER-TBI Investigator	
Roger	Lightfoot			Department of Anesthesiology & Inte	Southhampton, UK	CENTER-TBI Investigator	
Hester	Lingsma			Department of Public Health, Erasmu	Rotterdam, Netherlands	CENTER-TBI Participant + MC	
Marc	Maegele			Cologne-Merheim Medical Center (C	Cologne, Germany	CENTER-TBI Participant	
Marek	Majdan			Department of Public Health, Faculty	Trnava, Slovakia	CENTER-TBI Participant	
Alex	Manara			Intensive Care Unit, Southmead Hosp	Bristol, UK	CENTER-TBI Investigator	
Geoffrey	Manley			Department of Neurological Surgery,	San Francisco, California, U	CENTER-TBI Participant	
Hugues	Maréchal			Intensive Care Unit, CHR Citadelle , Li	Liège, Belgium	CENTER-TBI Investigator	
Costanza	Martino			Department of Anesthesia & Intensiv	Cesena, Italy	CENTER-TBI Investigator	
Julia	Mattern			Department of Neurosurgery, Univer	Heidelberg, Germany	CENTER-TBI Participant	

*Indicates required information. Only first name, last name, and suffix will appear in PubMed.

*First Name and Middle Initial(s)	*Last Name	*Suffix (eg, Jr, III)	Academic Degrees	Institution	Location (city, state/province, country)	Role or Contribution, eg, chair, principal investigator	Group (if more than 1 Group listed in the byline) and/or Subgroup (eg, Steering Committee)
Catherine	McMahon			Department of Neurosurgery, The Wa	Liverpool, UK	CENTER-TBI Investigator	
Béla	Melegh			Department of Medical Genetics, Uni	Pécs, Hungary	CENTER-TBI Participant	
Tomas	Menovsky			Department of Neurosurgery, Antwe	Edegem, Belgium	CENTER-TBI Participant + Investigator	
Ana	Mikolic			Department of Public Health, Erasmu	Rotterdam, Netherlands	CENTER-TBI Participant	
Benoit	Misset			Cyclotron Research Center , Universit	Liège, Belgium	CENTER-TBI Participant + Investigator	
Visakh	Muraleedharan			Karolinska Institutet, INCF Internatior	Stockholm, Sweden	CENTER-TBI Participant + DCTF	
Lynnette	Murray			ANZIC Research Centre, Monash Univ	Melbourne, Australia	Oz ENTER	
Nandesh	Nair			Department of Neurosurgery, Antwe	Antwerp, Belgium	CENTER-TBI Participant	
Ancuta	Negru			Department of Neurosurgery, Emerg	Timisoara, Romania	CENTER-TBI Investigator	
David	Nelson			Karolinska Institutet, INCF Internatior	Stockholm, Sweden	CENTER-TBI Participant + Investigator	
Virginia	Newcombe			Division of Anaesthesia, University of	Cambridge, UK	CENTER-TBI Participant + Investigator	
Daan	Nieboer			Department of Public Health, Erasmu	Rotterdam, Netherlands	CENTER-TBI Participant	
József	Nyirádi			János Szentágothai Research Centre,	Pécs, Hungary	CENTER-TBI Participant + Investigator	
Matej	Oresic			School of Medical Sciences, Örebro U	Örebro, Sweden	CENTER-TBI Participant	
Fabrizio	Ortolano			Neuro ICU, Fondazione IRCCS Cà G	Milan, Italy	CENTER-TBI Participant + Investigator	
Olubukola	Otesile			Centre for Urgent and Emergency	Sheffield, UK	CENTER-TBI Participant + Investigator	
Aarno	Palotie			Institute for Molecular Medicine Finla	Helsinki, Finland	CENTER-TBI Participant	
Paul M.	Parizel			Department of Radiology, University	Edegem, Belgium	CENTER-TBI Participant + Investigator	
Jean-François	Payen			Department of Anesthesiology & Inte	Grenoble, France	CENTER-TBI Investigator	
Natascha	Perera			International Projects Management,	Munchen, Germany	CENTER-TBI Participant	
Vincent	Perlbarg			Anesthesie-Réanimation, Assistance	Paris, France	CENTER-TBI Participant + Investigator	
Paolo	Persona			Department of Anesthesia & Intensiv	Padova, Italy	CENTER-TBI Investigator	
Wilco	Peul			Dept. of Neurosurgery, Leiden Univer	Leiden, Netherlands	CENTER-TBI Participant + Investigator + MC	
Anna	Piippo-Karjalainen			Department of Neurosurgery, Helsink	Helsinki, Finland	CENTER-TBI Investigator	
Matti	Pirinen			Institute for Molecular Medicine Finla	Helsinki, Finland	CENTER-TBI Participant	
Dana	Pisica			Department of Public Health, Erasmu	Rotterdam, Netherlands	CENTER-TBI Participant	
Horia	Ples			Department of Neurosurgery, Emerg	Timisoara, Romania	CENTER-TBI Investigator	
Suzanne	Polinder			Department of Public Health, Erasmu	Rotterdam, Netherlands	CENTER-TBI Participant	
Inigo	Pomposo			Department of Neurosurgery, Hospita	Bilbao, Spain	CENTER-TBI Investigator	
Jussi P.	Posti			Division of Clinical Neurosciences, De	Turku, Finland	CENTER-TBI Participant + Investigator	
Louis	Puybasset			Department of Anesthesiology and C	Paris, France	CENTER-TBI Participant + Investigator	

*Indicates required information. Only first name, last name, and suffix will appear in PubMed.

*First Name and Middle Initial(s)	*Last Name	*Suffix (eg, Jr, III)	Academic Degrees	Institution	Location (city, state/province, country)	Role or Contribution, eg, chair, principal investigator	Group (if more than 1 Group listed in the byline) and/or Subgroup (eg, Steering Committee)
Andreea	Rădoi			Neurotraumatology and Neurosurger	Barcelona, Spain	CENTER-TBI Investigator	
Arminas	Ragauskas			Department of Neurosurgery, Kaunas	Vilnius, Lithuania	CENTER-TBI Investigator	
Rahul	Raj			Department of Neurosurgery, Helsink	Helsinki, Finland	CENTER-TBI Investigator	
Malinka	Rambadagalla			Rezekne Hospital, Latvia	Rezekne, Latvia	CENTER-TBI Investigator	
Isabel	Retel Helmrich			Department of Public Health, Erasmu	Rotterdam, Netherlands	CENTER-TBI Participant	
Jonathan	Rhodes			Department of Anaesthesia, Critical C	Edinburgh, UK	CENTER-TBI Investigator	
Sylvia	Richardson			Director, MRC Biostatistics Unit, Cam	Cambridge, UK	CENTER-TBI Participant	
Sophie	Richter			Division of Anaesthesia, University of	Cambridge, UK	CENTER-TBI Participant + Investigator	
Samuli	Ripatti			Institute for Molecular Medicine Finla	Helsinki, Finland	CENTER-TBI Participant	
Saulius	Rocka			Department of Neurosurgery, Kaunas	Vilnius, Lithuania	CENTER-TBI Investigator	
Cecilie	Roe			Department of Physical Medicine and	Oslo, Norway	CENTER-TBI Participant + Investigator	
Olav	Roise			Division of Orthopedics, Oslo Univers	Oslo, Norway	CENTER-TBI Participant + Investigator	
Jonathan	Rosand			Broad Institute, Cambridge MA Harva	Boston, Massachusetts, US	CENTER-TBI Associated Participant	
Jeffrey	Rosenfeld			National Trauma Research Institute, T	Melbourne, Australia	CENTER-TBI Participant	
Christina	Rosenlund			Department of Neurosurgery, Odense	Odense, Denmark	CENTER-TBI Investigator	
Guy	Rosenthal			Department of Neurosurgery, Hadass	Jerusalem, Israel	CENTER-TBI Investigator	
Rolf	Rossaint			Department of Anaesthesiology, Univ	Aachen, Germany	CENTER-TBI Associated participant + Investigator	
Sandra	Rossi			Department of Anesthesia & Intensiv	Padova, Italy	CENTER-TBI Investigator	
Daniel	Rueckert			Department of Computing, Imperial C	London, UK	CENTER-TBI Participant	
Martin	Rusnák			International Neurotrauma Research	Vienna, Austria	CENTER-TBI Participant	
Juan	Sahuquillo			Department of Neurosurgery, Vall d'H	Barcelona, Spain	CENTER-TBI Investigator	
Oliver	Sakowitz			Department of Neurosurgery, Univer	Heidelberg, Germany	CENTER-TBI Participant	
Renan	Sanchez-Porras			Klinik für Neurochirurgie, Klinikum Lu	Ludwigsburg, Germany	CENTER-TBI Participant + Investigator	
Janos	Sandor			Division of Biostatistics and Epidemio	Debrecen, Hungary	CENTER-TBI Participant	
Nadine	Schäfer			Cologne-Merheim Medical Center (CM	Cologne, Germany	CENTER-TBI Participant	
Silke	Schmidt			Department Health and Prevention, U	Greifswald, Germany	CENTER-TBI Participant	
Herbert	Schoechl			Department of Anaesthesiology and I	Salzburg, Austria	CENTER-TBI Associated Participant	
Guus	Schoonman			Department of Neurology, Elisabeth-T	Tilburg, Netherlands	CENTER-TBI Investigator	
Rico Frederik	Schou			Department of Neuroanesthesia and	Odense, Denmark	CENTER-TBI Participant + Investigator	
Elisabeth	Schwendenwein			Trauma Surgery, Medical University V	Vienna, Austria	CENTER-TBI Investigator	
Ranjit D.	Singh			Dept. of Neurosurgery, Leiden Uni	Leiden, Netherlands	CENTER-TBI Investigator	

Supplemental Online Content: Nonauthor Collaborators

*Indicates required information. Only first name, last name, and suffix will appear in PubMed.

***First Name and Middle Initial(s)**	***Last Name**	***Suffix (eg, Jr, III)**	Academic Degrees	Institution	Location (city, state/province, country)	Role or Contribution, eg, chair, principal investigator	Group (if more than 1 Group listed in the byline) and/or Subgroup (eg, Steering Committee)
Charlie	Sewalt			Department of Public Health, Erasmu	Rotterdam, Netherlands	CENTER-TBI Participant	
Toril	Skandsen			Department of Neuromedicine and M	Trondheim, Norway	CENTER-TBI Investigator	
Peter	Smielewski			Brain Physics Lab, Division of Neurosu	Cambridge, UK	CENTER-TBI Participant + Investigator	
Abayomi	Sorinola			Department of Neurosurgery, Univer	Pécs, Hungary	CENTER-TBI Participant + Investigator	
Emmanuel	Stamatakis			Division of Anaesthesia, University of	Cambridge, UK	CENTER-TBI Participant	
Simon	Stanworth			Oxford University Hospitals NHS Trus	Oxford, UK	CENTER-TBI Participant	
Robert	Stevens			Division of Neuroscience Critical Care	Baltimore, Maryland, USA	CENTER-TBI Associated Participant	
Ewout W.	Steyerberg			Dept. of Department of Biomedical D	Leiden, Netherlands	CENTER-TBI Participant + MC	
Nino	Stocchetti			Department of Pathophysiology and T	Milan, Italy	CENTER-TBI Participant + Investigator + MC	
Nina	Sundström			Department of Radiation Sciences, Bi	Umea, Sweden	CENTER-TBI Investigator	
Riikka	Takala			Perioperative Services, Intensive Care	Turku, Finland	CENTER-TBI Participant + Investigator	
Viktória	Tamás			Department of Neurosurgery, Univer	Pécs, Hungary	CENTER-TBI Participant + Investigator	
Tomas	Tamosuitis			Department of Neurosurgery, Kaunas	Vilnius, Lithuania	CENTER-TBI Investigator	
Mark Steven	Taylor			Department of Public Health, Faculty	Trnava, Slovak Republic	CENTER-TBI Participant	
Braden	Te Ao			National Institute for Stroke and App	Auckland, New Zealand	CENTER-TBI Participant	
Olli	Tenovuo			Division of Clinical Neurosciences, De	Turku, Finland	CENTER-TBI Participant + Investigator	
Alice	Theadom			National Institute for Stroke and App	Auckland, New Zealand	CENTER-TBI Participant	
Matt	Thomas			Intensive Care Unit, Southmead Hosp	Bristol, UK	CENTER-TBI Investigator	
Aurore	Thibaut			Cyclotron Research Center , Universit	Liège, Belgium	CENTER-TBI Participant	
Dick	Tibboel			Intensive Care and Department of Pe	Rotterdam, Netherlands	CENTER-TBI Participant	
Marjolijn	Timmers			Department of Intensive Care and De	Rotterdam, Netherlands	CENTER-TBI Participant	
Christos	Tolias			Department of Neurosurgery, Kings c	London, UK	CENTER-TBI Investigator	
Tony	Trapani			ANZIC Research Centre, Monash Univ	Melbourne, Australia	Oz ENTER	
Cristina Maria	Tudora			Department of Neurosurgery, Emerge	Timisoara, Romania	CENTER-TBI Investigator	
Andreas	Unterberg			Department of Neurosurgery, Univer	Heidelberg, Germany	CENTER-TBI Participant + Investigator	
Peter	Vajkoczy			Neurologie, Neurochirurgie und Psycl	Berlin, Germany	CENTER-TBI Investigator	
Egils	Valeinis			Neurosurgery clinic, Pauls Stradins Cl	Riga, Latvia	CENTER-TBI Investigator	
Shirley	Vallance			ANZIC Research Centre, Monash Univ	Melbourne, Australia	Oz ENTER	
Zoltán	Vámos			Department of Anaesthesiology and I	Pécs, Hungary	CENTER-TBI Participant + Investigator	
Mathieu	van der Jagt			Department of Intensive Care Adults,	Rotterdam, Netherlands	CENTER-TBI Investigator	
Joukje	van der Naalt			Department of Neurology, University	Groningen, Netherlands	CENTER-TBI Investigator	

Supplemental Online Content: Nonauthor Collaborators

*Indicates required information. Only first name, last name, and suffix will appear in PubMed.

***First Name and Middle Initial(s)**	***Last Name**	***Suffix (eg, Jr, III)**	Academic Degrees	Institution	Location (city, state/province, country)	Role or Contribution, eg, chair, principal investigator	Group (if more than 1 Group listed in the byline) and/or Subgroup (eg, Steering Committee)
Gregory	Van der Steen			Department of Neurosurgery, Antwer	Edegem, Belgium	CENTER-TBI Participant	
Jeroen T.J.M.	van Dijck			Dept. of Neurosurgery, Leiden Univer	Leiden, Netherlands	CENTER-TBI Participant + Investigator	
Inge A.	van Erp			Dept. of Neurosurgery, Leiden Uni	Leiden, Netherlands	CENTER-TBI Investigator	
Thomas A.	van Essen			Dept. of Neurosurgery, Leiden Univer	Leiden, Netherlands	CENTER-TBI Participant + Investigator	
Wim	Van Hecke			icoMetrix NV, Leuven, Belgium	Leuven, Belgium	CENTER-TBI Participant	
Caroline	van Heugten			Movement Science Group, Faculty of	Oxford, UK	CENTER-TBI Participant	
Dominique	Van Praag			Psychology Department, Antwerp Un	Edegem, Belgium	CENTER-TBI Participant + Investigator	
Ernest	van Veen			Department of Public Health, Erasmu	Rotterdam, Netherlands	CENTER-TBI Participant	
Roel P.J.	van Wijk			Dept. of Neurosurgery, Leiden Univer	Leiden, Netherlands	CENTER-TBI Participant + Investigator	
Thijs	Vande Vyvere			icoMetrix NV, Leuven, Belgium	Edegem, Belgium	CENTER-TBI Participant + Investigator	
Alessia	Vargiolu			NeuroIntenisve Care Unit, Departme	Monza, Italy	CENTER-TBI Participant + Investigator	
Emmanuel	Vega			Department of Anesthesiology-Intens	Lille, France	CENTER-TBI Investigator	
Kimberley	Velt			Department of Public Health, Erasmu	Rotterdam, Netherlands	CENTER-TBI Participant	
Jan	Verheyden			icoMetrix NV, Leuven, Belgium	Leuven, Belgium	CENTER-TBI Participant	
Paul M.	Vespa			Director of Neurocritical Care, Univer	Los Angeles, California, US/	CENTER-TBI Associated Participant	
Anne	Vik			Department of Neuromedicine and N	Trondheim, Norway	CENTER-TBI Investigator	
Rimantas	Vilcinis			Department of Neurosurgery, Kaunas	Kaunas, Lithuania	CENTER-TBI Investigator	
Victor	Volovici			Department of Neurosurgery, Erasmu	Rotterdam, Netherlands	CENTER-TBI Participant + Investigator	
Nicole	von Steinbüchel			Institute of Medical Psychology and N	Göttingen, Germany	CENTER-TBI Participant	
Daphne	Voormolen			Department of Public Health, Erasmu	Rotterdam, Netherlands	CENTER-TBI Participant	
Peter	Vulekovic			Department of Neurosurgery, Clinical	Novi Sad, Serbia	CENTER-TBI Investigator	
Kevin K.W.	Wang			Department of Emergency Medicine,	Gainesville, Florida, USA	CENTER-TBI Participant	
Daniel	Whitehouse			Division of Anaesthesia, University	Cambridge, UK	CENTER-TBI Investigator	
Eveline	Wiegers			Department of Public Health, Erasmu	Rotterdam, Netherlands	CENTER-TBI Participant	
Guy	Williams			Division of Anaesthesia, University of	Cambridge, UK	CENTER-TBI Participant	
Stefan	Winzeck			Division of Anaesthesia, University	Cambridge, UK	CENTER-TBI Investigator	
Stefan	Wolf			Interdisciplinary Neuro Intensive Care	Berlin, Germany	CENTER-TBI Participant + Investigator	
Zhihui	Yang			Department of Emergency Medicine,	Gainesville, Florida, USA	CENTER-TBI Participant	
Peter	Ylén			VTT Technical Research Centre, Tamp	Tampere, Finland	CENTER-TBI Participant	
Alexander	Younsi			Department of Neurosurgery, Univer	Heidelberg, Germany	CENTER-TBI Investigator	
Frederick A.	Zeiler			Division of Anaesthesia, University of	Cambridge, UK	CENTER-TBI Participant	

Supplemental Online Content: Nonauthor Collaborators

*Indicates required information. Only first name, last name, and suffix will appear in PubMed.

***First Name and Middle Initial(s)**	***Last Name**	***Suffix (eg, Jr, III)**	Academic Degrees	Institution	Location (city, state/province, country)	Role or Contribution, eg, chair, principal investigator	Group (if more than 1 Group listed in the byline) and/or Subgroup (eg, Steering Committee)
Veronika	Zelinkova			Department of Public Health, Facu	Trnava, Slovakia	CENTER-TBI Investigator	
Agate	Ziverte			Neurosurgery clinic, Pauls Stradins Cl	Riga, Latvia	CENTER-TBI Investigator	
Tommaso	Zoerle			Neuro ICU, Fondazione IRCCS Cà G	Milan, Italy	CENTER-TBI Participant + Investigator	
