# Supplementary material for: Contrasting Characteristics and Outcomes of Sports-Related and Non–Sports-Related Traumatic Brain Injury
Source: JAMA Netw Open. 2024 Jan 24;7(1):e2353318. doi: 10.1001/jamanetworkopen.2023.53318 (PMC10809021; doi:10.1001/jamanetworkopen.2023.53318)
Supplement: Supplement 3. — Data Sharing Statement [file jamanetwopen-e2353318-s003.pdf]

Data Sharing Statement

Ntikas. Contrasting Characteristics and Outcomes of Sports- and Non–Sports-Related Traumatic Brain Injury. *JAMA Netw Open*. Published January 31, 2024. doi:10.1001/jamanetworkopen.2023.53318

Data
Data available: Yes
Data types: Deidentified participant data
How to access data: Individual patient data is available by application to CENTER-TBI (https://www.center-tbi.eu/data).
When available: beginning date: 09-08-2023

Supporting Documents
Document types: None

Additional Information
Who can access the data: Data will be made available to researchers whose proposed use of the data has been approved by the CENTER-TBI Management Committee
Types of analyses: Data will be made available for the purpose of the proposed analysis.
Mechanisms of data availability: After approval of a proposal and with a data access agreement
